# Expression profile of microRNAs in the testes of patients with Klinefelter syndrome

**DOI:** 10.1038/s41598-020-68294-7

**Published:** 2020-07-10

**Authors:** Marisol Ibarra-Ramírez, Geovana Calvo-Anguiano, José de Jesús Lugo-Trampe, Laura Elia Martínez-de-Villarreal, David Rodríguez-Torres, Manuel Nistal, Pilar González-Peramato

**Affiliations:** 1 Genetics Department, Faculty of Medicine and University Hospital “José E. González”, Universidad Autónoma de Nuevo León, Av. Gonzalitos s/n cruce con Av. Madero, Col. Mitras Centro, CP 64460 Monterrey, N.L México; 2 Department of Anatomy, Histology and Neurosciences, Universidad Autónoma de Madrid, C/Arzobispo Morcillo 4, CP 28029 Madrid, España; 3 Department of Pathology Anatomy, University Hospital “La Paz” Y Universidad Autónoma de Madrid, C/Arzobispo Morcillo 4, CP 28029 Madrid, España

**Keywords:** Disease genetics, Genetics research

## Abstract

Klinefelter syndrome (KS) is the most common sex chromosome aneuploidy. A distinctive characteristic of KS is oligozoospermia. Despite multiple studies that have described the natural history of the degenerative process of germ cells in patients with KS, the molecular mechanisms that initiate this process are not well characterized. MicroRNA (miRNA)-mediated post-transcriptional control mechanisms have been increasingly recognized as important regulators of spermatogenesis; however, only a few studies have evaluated the role of miRNAs in the gonadal failure of these patients. Here, we describe a differential expression profile for the miRNAs in testicular tissue samples taken from KS patients. We analysed testicular tissue samples from 4 KS patients and 5 control patients (obstructive azoospermia) through next-generation sequencing, which can provide information about the mechanisms involved in the degeneration of germ cells. A distinctive differential expression profile was identified for 166 miRNAs in the KS patients: 66 were upregulated, and 100 were downregulated. An interactome analysis was performed for 7 of the upregulated and the 20 downregulated miRNAs. The results showed that the target genes are involved in the development, proliferation, and differentiation processes of spermatogenesis, which may explain their role in the development of infertility. This is the first report of a miRNA expression profile generated from testicular tissue samples of KS patients.

## Introduction

Klinefelter syndrome (KS) is the most common sex chromosome aneuploidy in humans, with a prevalence of 1:500 live births^1^. It was first described in 1942 as a syndrome characterized by gynaecomastia, azoospermia, and elevated levels of follicle-stimulating hormone (FSH)^2^. The presence of an extra X chromosome (47,XXY) in the standard male chromosome set (46,XY) causes Klinefelter syndrome. Chromosomal nondisjunction during meiosis or early embryonic mitosis causes this aneuploidy. Cytogenetic confirmation of aneuploidy is performed through conventional karyotype analysis. Approximately 85–90% of KS patients present with a 47,XXY chromosome set, while the remaining 10–15% of patients present with mosaicism (47,XXY/46,XY) or aneuploidies with more than three sex chromosomes (48,XXXY or 48,XXYY)^3,4^.

Sexual development in KS patients may be healthy before puberty, showing typical pubertal changes and levels of luteinizing hormone (LH), FSH, and testosterone^5^. During adolescence, the testicles appear small and firm and the patient presents with multiple symptoms of androgen deficiency. Hypogonadotropic hypogonadism (HH) is the predominant characteristic, with testosterone levels below 12 nmol/L; low levels of insulin-like 3 (INSL 3), inhibin B and anti-Müllerian hormone (AMH); and high levels of FSH and LH^6^. Infertility is one of the distinctive characteristics of KS, found in approximately 10% of azoospermia patients. During puberty, the testicles of KS patients grow to a volume of 6 ml; however, although the serum levels of testosterone increase, the following features appear: decreasing the number of germ cells, hyaline seminiferous tubules, degenerated Sertoli cells, and hyperplasic Leydig cells^7,8^.

Histological analysis of adult testes of KS patients shows abundant seminiferous tubule hyalinization and interstitial Leydig cell pseudohyperplasia; some studies also reported findings of Sertoli cell-only tubules^9^. Although semen analyses of adult KS patients have revealed azoospermia at high frequency, some men with KS may have residual spermatogenesis foci; approximately 7.7–8.4% of KS patients produce spermatozoa^10^. Unfortunately, natural conception in KS-related infertile couples is very rare; most often, the only hope for biological paternity is through testicular sperm extraction (TESE) combined with intracytoplasmic sperm injection (ICSI). A meta-analysis of data on surgical sperm retrieval rates showed the presence of spermatozoa in approximately 40% of the KS patients who underwent TESE^10,11^. Nevertheless, the reproductive options for these patients are limited and reduced further because the subjacent mechanisms leading to germ cell degeneration are unknown.

Mouse models with a 41,XXY chromosome set show regular germ cell migration and early testis development, but germ cell number expansion decreases as development proceeds. Transplantation of XY germ cells in 41,XXY testes enables some cells to complete spermatogenesis, suggesting that the primary cause of spermatogenesis disruption can be found in germ cells^12^.

Spermatogenesis is a strictly regulated process at the transcriptional and post-transcriptional levels leading to the continuous production of gametes during adulthood. The post-transcriptional control mechanism mediated by microRNAs (miRNAs) is essential for spermatogenesis regulation^13^. miRNAs are small (22 nucleotides), nuclear DNA-encoded, non-coding RNA molecules produced in eukaryotic cells. In general, miRNAs silence gene expression by targeting mRNA for degradation or by interfering with its translation^14,15^; however, few studies have investigated the role of miRNAs in the phenotype of patients with KS^16,17^.

Transcriptional silencing of genes on X and Y chromosomes occurs in the second half of the pachytene phase; however, most of the miRNAs associated with the X chromosome are transcribed and processed at this stage. Approximately 86% of the miRNAs located on the X chromosome escape meiotic inactivation during spermatogenesis^18^. This finding suggests that the levels of these miRNAs may be abnormally high in 47,XXY testes, potentially leading to the misregulation of autosomal mRNAs during meiosis and spermatogenesis disruption in KS patients. This work was based on the hypothesis that the expression profile of miRNAs in the testicular tissue of Klinefelter patients differs from that of the controls and at least some of the target genes for differentially expressed miRNAs could be involved in spermatogenesis.

## Results

Seven (7) testicular samples from 4 Klinefelter syndrome patients and five (5) control testicular samples from 5 patients with obstructive azoospermia were analysed. The patients’ age range was 33–36 years old. One of the patients had a mosaic 47,XXY/46,XY chromosome set (KLI_5 and KLI_6). All the control samples were obtained from biopsied tissue of patients with infertility because of azoospermia who were in an age range of 30–40 years. All the control patients had a 46,XY karyotype.

## Profiling differentially expressed miRNAs

**Figure 1:**
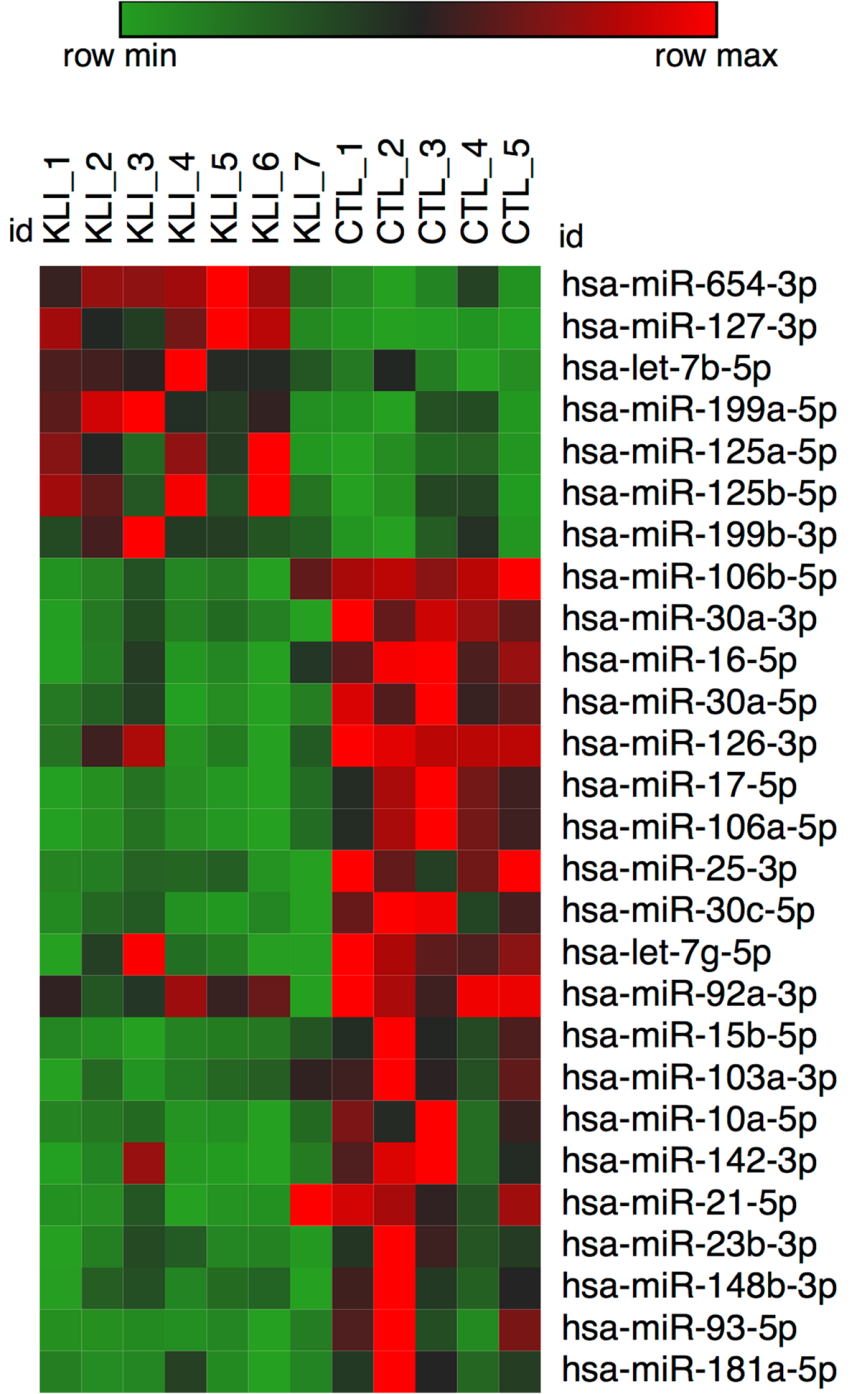
Heatmap of differentially expressed miRNAs**.** Heatmap depicting the differential expression of miRNAs in the Klinefelter and control testicular tissue samples. Twenty (20) of these miRNAs showed downregulated expression (red), and 7 showed upregulated expression (green) in the samples from the Klinefelter syndrome patients compared to the levels in the samples from the control group.

**Figure 2:**
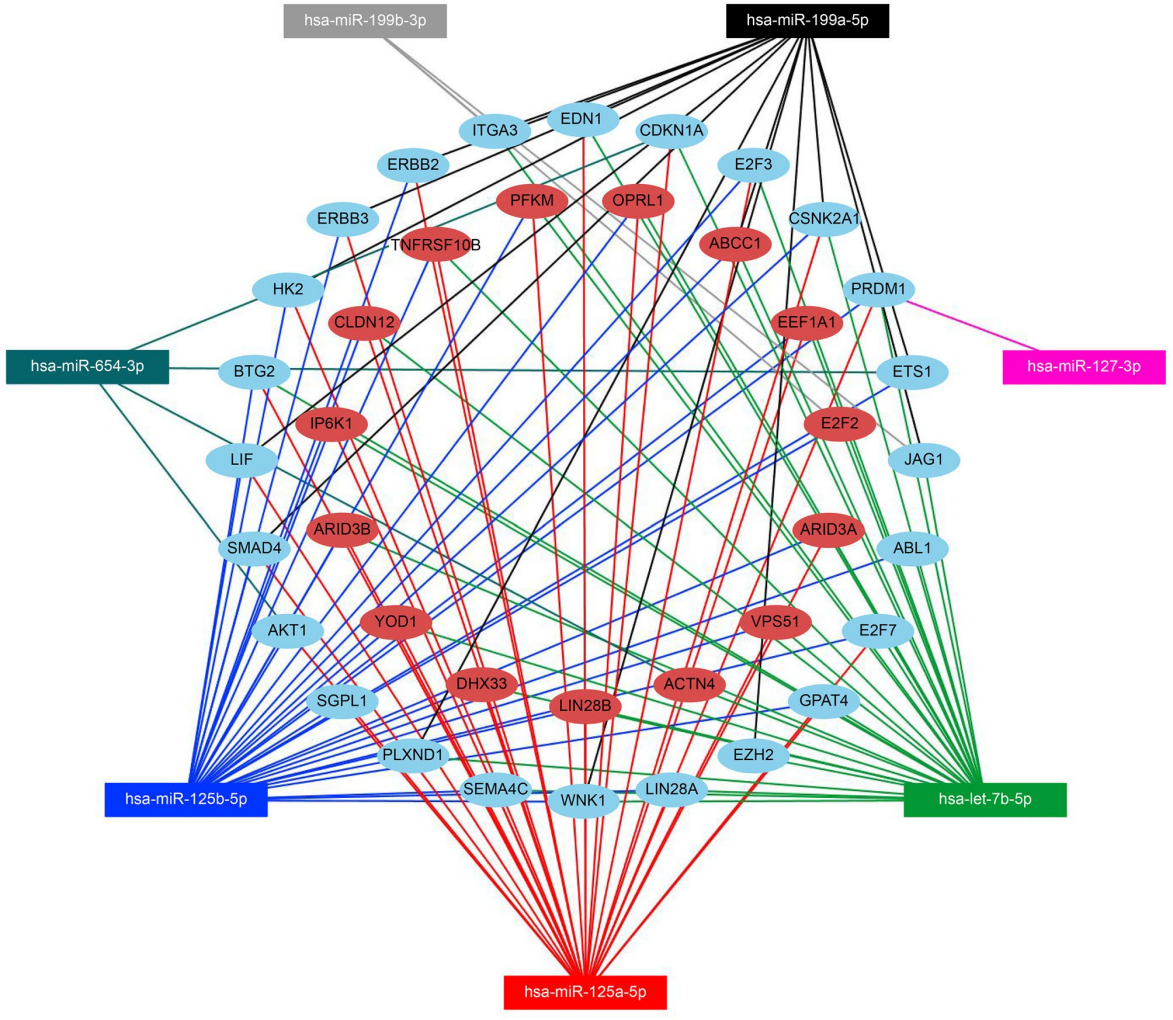
miRnet interactome network for the high-expression, upregulated miRNAs in the KS sample subset. Interaction between highly expressed upregulated miRNAs, hsa-miR-654-3p, hsa-miR-127-3p, hsa-let-7b-5p, hsa-miR-199a-5p, hsa-miR-125a-5p, hsa-miR-125b-5p, and hsa-miR-199b-3p (squares), in the Klinefelter sample tissues with target genes (blue and red dots). Blue dots represent genes involved in different cell functions: differentiation, cell proliferation, system development, and reproduction.

Using a significance threshold of *p* < 0.05, one hundred and sixty-six (166) hsa-miRNAs with differential expression in the KS patients and controls were found and were composed of 100 downregulated and 66 upregulated miRNA transcripts in the KS sample (Table S1, Supplementary data). Of the 166 miRNAs that showed differential expression, 27 belonged to the high-expression level group (i.e., number of sequence counts above the average); of these, 20 were downregulated and 7 were upregulated in the KS samples in relation to their expression levels in the control group (Table 1 and Fig. 1). The seven (7) upregulated, and the 20 downregulated miRNAs were used in separate interactome analyses using the miRNet database^19,20^ since they are likely to interfere with gene expression of their target genes. The miRNA target prediction was followed by an interactome network analysis and a Gene Ontology (GO) enrichment for the following terms: gamete generation, positive regulation of cell differentiation, cell morphogenesis involved in differentiation, developmental maturation, cell proliferation, and reproductive sexual process. This process produced a group of 39 target genes for the 7 upregulated miRNAs (Fig. 2 and Supplementary data Table S2), and 70 target genes for the 20 downregulated miRNAs (Supplementary data Figure S1 and Table S3).

**Table 1:** List of differentially expressed miRNAs.

miR_name	Up/down	log2 (fold_change)	*p* Value (t_test)
hsa-miR-654-3p	Up	1.84	8.72E−04
hsa-miR-127-3p	Up	2.67	2.18E−03
hsa-let-7b-5p	Up	0.78	8.25E−03
hsa-miR-199a-5p	Up	1.35	1.37E−02
hsa-miR-125a-5p	Up	1.27	1.76E−02
hsa-miR-125b-5p	Up	0.98	1.79E−02
hsa-miR-199b-3p	Up	1.03	3.78E−02
hsa-miR-106b-5p	Down	− 1.23	7.75E−05
hsa-miR-30a-3p	Down	− 1.39	9.56E−05
hsa-miR-16-5p	Down	− 1.29	3.62E−04
hsa-miR-30a-5p	Down	− 1.39	9.21E−04
hsa-miR-126-3p	Down	− 0.93	1.22E−03
hsa-miR-17-5p	Down	− 1.90	1.58E−03
hsa-miR-106a-5p	Down	− 1.90	1.58E−03
hsa-miR-25-3p	Down	− 1.16	5.13E−03
hsa-miR-30c-5p	Down	− 0.97	5.49E−03
hsa-let-7g-5p	Down	− 0.71	8.96E−03
hsa-miR-92a-3p	Down	− 0.62	1.01E−02
hsa-miR-15b-5p	Down	− 1.56	1.29E−02
hsa-miR-103a-3p	Down	− 0.58	1.90E−02
hsa-miR-10a-5p	Down	− 1.75	2.22E−02
hsa-miR-142-3p	Down	− 1.57	2.74E−02
hsa-miR-21-5p	Down	− 0.96	2.78E−02
hsa-miR-23b-3p	Down	− 0.99	2.85E−02
hsa-miR-148b-3p	Down	− 0.63	3.44E−02
hsa-miR-93-5p	Down	− 1.80	3.96E−02
hsa-miR-181a-5p	Down	− 0.78	4.01E−02

There were 27 miRNAs in the high-expression group that displayed differential expression (up/down) in the KS and control samples. From the 166 miRNAs that showed differential expression, we showed only those that were either highly upregulated- or downregulated and listed them according to significance.

The seven (7) upregulated miRNAs were: hsa-miR-125a-5p, hsa-miR-125b-5p, hsa-let-7b-5p, hsa-miR-199a-5p, hsa-miR-199b-3p, hsa-miR-654-3p, and hsa-miR-127-3p.The target genes of these upregulated miRNAs are involved in the following biological processes: cell proliferation (*JAG1, PRDM1, CDKN1A, E2F3, EDN1, BTG2, E2F7, ERBB2, ERBB3, ETS1, LIF, SMAD4,* and *AKT1*), positive regulation of cell differentiation (*ABL1, JAG1, PRDM1, EDN1, EZH2, LIN28A, ETS1, LIF, SMAD4,* and *AKT1),* positive regulation of developmental processes (*ABL1, JAG1, PRDM1, CDKN1A, CSNK2A1, EDN1, ITGA3, BTG2, PLXND1, SEMA4C, WNK1, EZH2, LIN28A, GPAT4, E2F7, ERBB2, ERBB3, ETS1, HK2, LIF, SMAD4,* and *AKT1*) and reproductive processes and gamete generation (*PRDM1, EDN1, LIN28A, GPAT4, ETS1, HK2, LIF, AKT1,* and *SGPL1*). Some target genes participate in multiple and overlapped biological processes (Supplementary data figure S2). The target genes of the 20 downregulated miRNAs (hsa-miR-106b-5p, hsa-miR-30a-3p, hsa-miR-16-5p, hsa-miR-30a-5p, hsa-miR-126-3p, hsa-miR-17-5p, hsa-miR-106a-5p, hsa-miR-25-3p, hsa-miR-30c-5p, hsa-let-7g-5p, hsa-miR-92a-3p, hsa-miR-15b-5p, hsa-miR-103a-3p, hsa-miR-10a-5p, hsa-miR-142-3p, hsa-miR-21-5p, hsa-miR-23b-3p, hsa-miR-148b-3p, hsa-miR-93-5p and hsa-miR-181a-5p) are also involved in cell proliferation, regulation of developmental processes, regulation of cell differentiation, germ cell development, and gonadal development (*ACVR2A, CCND1, CCNE2, HMGA2, BMI1, HSP90AA1, NOTCH2, NR2C2* and *BCL2*).

**Figure 3:**
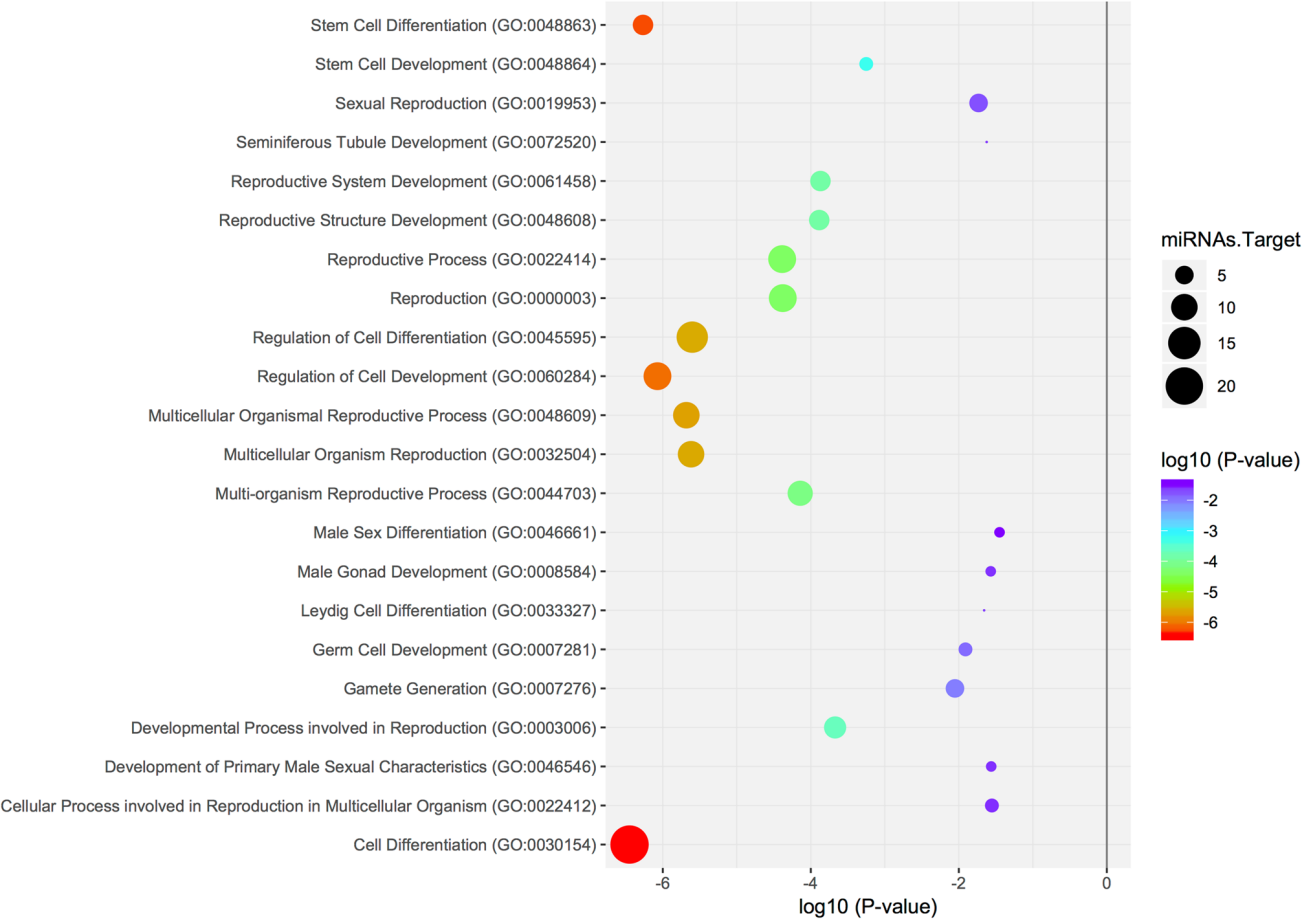
Scatterplot of enriched GO terms. This graphic represents the target genes of the upregulated miRNAs (7) enriched in GO terms, where the colour of the circle indicates the statistical significance expressed in log10 values and the size of the circle the number of target genes involved.

**Figure 4:**
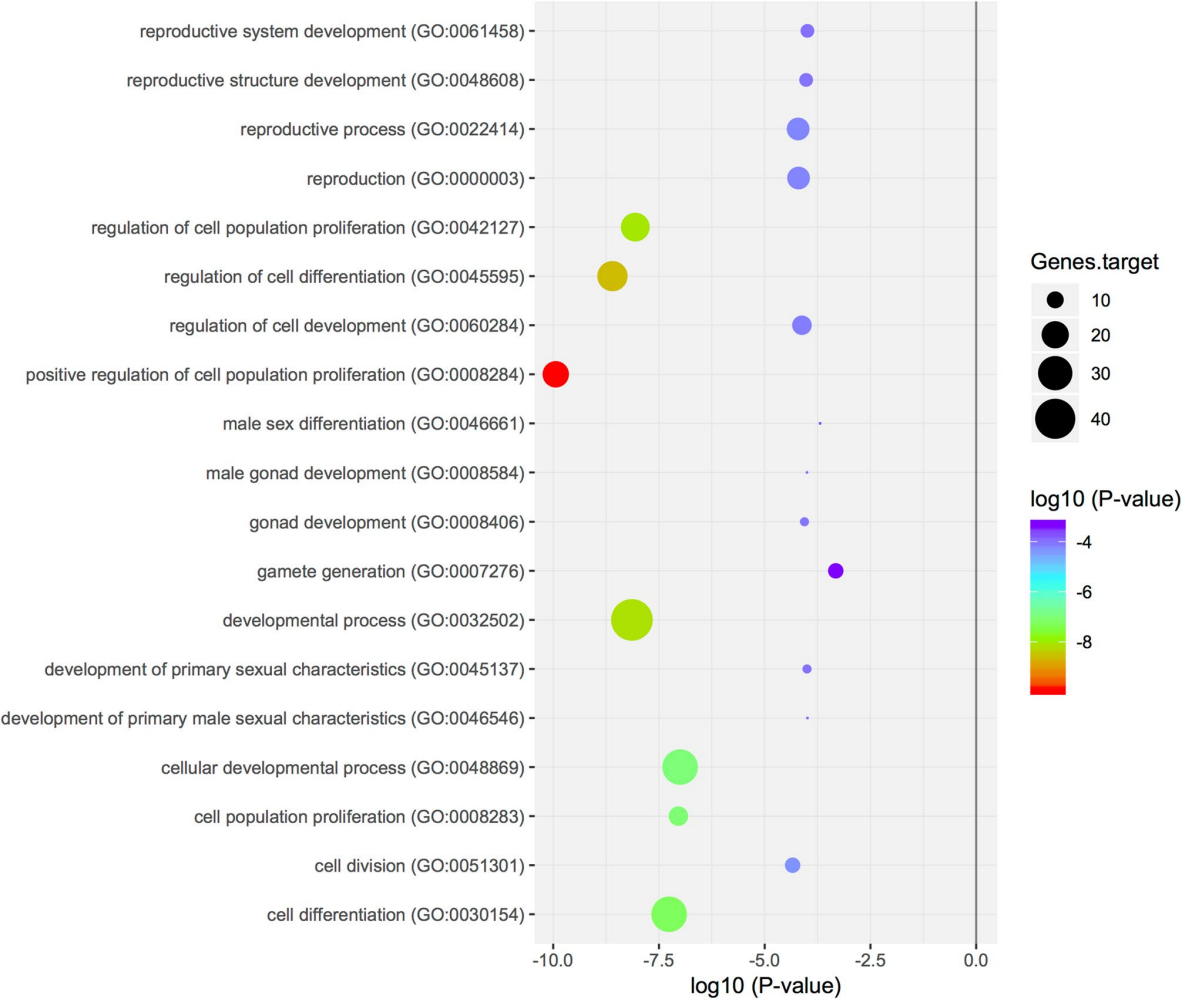
Scatterplot of enriched GO terms. This graphic represents the target genes of the downregulated miRNAs (20) enriched in GO terms, where the colour of the circle indicates the statistical significance expressed in log10 values and the size of the circle the number of target genes involved.

To perform statistical analysis on the biological processes for which the target genes have differentially expressed miRNAs, we carried out an over-representation test calculation (PANTHER platform) that yielded significant values (*p* < 0.05) for biological processes related to development, differentiation, and processes implicated in spermatogenesis (Data is shown in Figs. 3 and 4; Supplementary data tables S4 and S5). The participation of target genes in these biological processes is not exclusive as significant differences were also observed in others (for example, regulation of metabolic process (GO:0050789), regulation of gene expression (GO:0010468), regulation of developmental process (GO:0050793), and apoptotic process (GO:0006915)).

## Discussion

Noncoding RNAs play critical roles in the control of gene expression, and they are differentially regulated during spermatogenesis^21,22^. The roles of miRNAs as regulatory factors that control the expression of a large number of protein-coding genes are well documented. miRNAs function mainly after transcription, affecting the stability or translational efficiency of their target mRNAs^23^. Increased expression levels of miRNA genes located on the X chromosome may contribute to the KS phenotype. Here, miRNA expression was analysed in testicular tissue biopsy samples from patients with KS and control samples from patients with obstructive azoospermia with normal testicular tissue histology. Next-generation sequencing analysis enabled the construction of a differential expression profile. Previously, a miRNA cluster associated with Xq27 was shown to display testis-specific expression in healthy tissues (hsa-mir-506, hsa-mir-507, hsa-mir-508, hsa-mir-509, hsa-mir-510, hsa-mir-513, and hsa-mir-514)^24^. The expression of these miRNAs was detected in the present study in the testicular tissue from both experimental groups, which indicates that our approach provides reliable information regarding the miRNA expression profile. Interestingly, the expression levels for these specific miRNAs showed no differences between the normal males and KS patients.

The investigation of miRNAs revealed that a single miRNA can have multiple target mRNAs and different sets of miRNAs expressed in different cell types and tissues with multiple functions in the development of many biological processes. Abnormal miRNA expression is implicated in disease states^25^. An analysis of miRNA expression profiles in testicular tissue of male patients with infertility was previously published^26,27^. The first report of altered miRNA expression in the testes of patients with nonobstructive azoospermia was published by Lian et al., who showed a decrease in the expression of 154 miRNAs and a clear increase in the expression of 19 miRNAs^27^. Wu et al. evaluated the expression patterns of miR-i9B and let-7a in individuals with idiopathic infertility by quantitative PCR. They clearly showed that these two miRNAs were expressed at higher levels in the cases of infertile patients than they were in fertile individuals^28^.

Several miRNAs have been shown to play roles in multiple spermatogenesis stages. miRNAs follow phase-specific expression patterns inside the testis and at distinct phases of differentiation^22^. We were interested in analysing whether the miRNAs have a differential expression pattern among our experimental groups.

The mouse *Mirlet7* miRNA plays a critical role in the proliferation and differentiation of spermatogonial cells through the regulation of key genes^29^. Studies in a mouse Klinefelter model (41,XXY), in which the expression of *LIN28A* is low, suggested that azoospermic individuals are born with a reduced number of spermatogonia and consequently have a reduced number of spermatogonia throughout life^30^. *LIN28A* is an RNA-binding protein that is highly expressed in pluripotent mouse embryonic stem cells and is specifically expressed in undifferentiated mouse spermatogonia^31^. The expression of *LIN28A* is epigenetically regulated by the miR-125a and miR-125b. An increase in miR-125b resulted in the negative regulation of *Lin28A* mRNA. In the current study, a significant increase in the expression levels of hsa-mir-125b and hsa-mir-125a was observed in the KS samples compared to those of the control samples. The induction of let-7 by the downregulation of *LIN28A* inhibited the self-renewal capacity of non-differentiated cells and promoted their differentiation^32–34^. Here, there was a significant increase in the expression of hsa-let-7b miRNA. There was a significant difference in hsa-let-7g expression between groups, but in this case, the expression was lower in the KS patient samples.

Several miRNAs have been implicated in the regulation of spermatogonial stem cells (SSCs), including miR-21, miR-20, and miR-106a, which are preferentially expressed by SSCs^35^. Interestingly, the inhibition of miR-21 increased the number of germ cells that underwent apoptosis. In our study, we detected a decreased expression level of hsa-mir-21, which could have been related to the apoptosis of germ cells. Overexpression of miR-20 and miR-106 increased the proliferation rate of SSCs^36^. In our study, both of these miRNAs were expressed at a low level, with a significant decrease in the KS patient samples compared to their levels in the control samples.

Microarray and qPCR analyses have shown that cluster miR-449 miRNAs, miR34b, and miR-34c are preferentially expressed in mouse testes, and their expression levels increase at the beginning of meiosis during testicular development and adult spermatogenesis. The expression pattern of the miR-449 group is similar to that of miRNA-34b/c during spermatogenesis, which led to the suggestion that both clusters function in a redundant manner in the regulation of male germ cell development^37^. Our results showed a decrease in the expression levels of miR-34c, miR-34b, and interestingly, miR-449c and miR-449b expression was not detectable in KS patient samples. Decreased expression levels of miRNA-34b/c and miRNA-449 were reported for samples of infertile individuals^38^. An analysis of the effect of both increased expression level and inhibition of miR-34c on the expression of Nanos2 in GC-1 cells showed that abnormal expression of miR-34c/Nanos2 interrupted the balance between autorenewal and differentiation of SSCs and ultimately led to a negative effect on spermatogenesis in testes with cryptorchidism^39^.

The interactome network and miRNA target prediction analysis performed with the differentially expressed miRNAs found in this study showed a group of genes associated with biological processes that could be involved in mechanisms that affect spermatogenesis in KS patients. The results of an over-representation test showed significant differences in the processes of differentiation, development, and maintenance of germ cells and in reproductive processes, according to GO enrichment terms. Transcriptome analysis of the testicular tissue of a KS patient indicated a downregulated gene cluster of factors involved in spermatogenesis and male germ cell maturation and morphology^40^. A separate transcriptional analysis of testicular tissue from KS patients with hypospermatogenesis^41^ showed a downregulated gene cluster of factors involved in sperm production, including *EZH2*. This gene has a role in the maintenance of primordial germ cells. Interestingly, our work showed that miR-199a-5p and let-7b-5p, which target *EZH2,* were upregulated in the KS patient samples, indicating a possible decrease in *EZH2* expression.

Among other target genes of the upregulated miRNAs, the *ABL1* proto-oncogene, which encodes cytoplasmic and nuclear protein tyrosine kinases, is implicated in cell differentiation, cell division, cell adhesion, and stress-response processes^42^. Homozygous *Abl1*-mutant mice had reduced viability at birth, reduced fertility, shortened skull, and defects in the maturation of B cells in bone marrow^43^. A recent meta-analysis of gene expression profiles generated from testicular samples of humans with altered fertility suggested that *ABL1* was among the candidate genes associated with arrested spermatogenesis. It is possible that the function of the gene product(s) is mediated by cell cycle defects, but the actual mechanism is unknown^44^.

Jagged-1 (*JAG1*) is a ligand for the Notch receptor and its binding triggers a proteolytic excision cascade that eventually leads to the release of the intracellular part of the receptor from the membrane, which enables it to translocate to the nucleus where it activates transcription factors that play important roles in cell differentiation and morphogenesis^45^. JAG1 is also a target gene for miR-34a and miR-21, which are also implicated in the regulation of spermatogonial cell differentiation^35,46^. A separate study indicated that germ cells in mouse testes induce the activation of Notch in Sertoli cells through Jag1 and trigger their own differentiation through the negative regulation of *Cyp26b1.* When germ cells do not produce functional Jag1, the Notch pathway is not activated, and the germ cells remain in an undifferentiated state^47^. Interestingly, *NOTCH2,* a *NOTCH* receptor important during Leydig cell differentiation^48^, is a target gene of some of the downregulated miRNAs (hsa-mir-16-5p, hsa-mir-17-5p, hsa-mir-92a-3p, hsa-mir-106a-5p, hsa-mir-181a-5p, hsa-mir-15b-5p, and hsa-mir-23b-3p).

*ERBB2* encodes an epidermal growth factor tyrosine kinase that does not have its own ligand-binding domain and therefore does not bind to growth factors; however, it binds strongly to other members of the EGF receptor family to form heterodimers that stabilize ligand binding and increase the kinase-mediated activation of signalling pathways^47,49^. ERBB2 and ERBB3 were detected in undifferentiated type A spermatogonia and in a rat spermatogonial stem cell line. The diversity of receptors and ligands in the EGF superfamily in rat testis cells was analysed, and evidence for the role of ERBB3 and ERBB2 in the retinoic acid-induced differentiation of spermatogonia was obtained^50^.

Leukaemia inhibitory factor (*LIF*) is a pleiotropic cytokine that belongs to the interleukin 6 (IL6) family. It plays an important role in the balance mechanism between stem cell proliferation, and differentiation. In Sertoli cells, elimination of LIF receptor (*LIFR*) leads to a degenerative phenotype characterized by abnormal loss of germ cells, sperm stasis, distension, and posterior atrophy of the seminiferous tubules^51^.

Some target genes of the downregulated miRNAs, such as *ACVR2A, CCND1, CCNE2, HMGA2, BMI1, NR2C2* and *BCL2,* have been associated with gonadal development, and germ cell maintenance through different pathways which could be relevant to the molecular mechanisms affected in KS patients with azoospermia. The *ACVR2A, CCNE2*, and *HMGA2* genes participate in meiotic regulation during male germ cell development^52–54^. *NR2C2,* a member of the nuclear hormone receptor superfamily is an important factor in normal spermatogenesis; *NR2C2* knockout mice show defects in the late-stage pachytene and diplotene spermatocytes preventing progress and completion of the meiotic divisions^55^. *BMI1* plays important roles in maintaining germ stem cells and in regenerating spermatogenic progenitors after injury^56^.

It has been shown that Bcl-2 inhibits apoptosis of spermatogonia and the growth of spermatogonial stem cells in a cell-intrinsic manner^57^. The Bcl-2 family of genes encodes a family of closely-related proteins that possess either proapoptotic or antiapoptotic activity^58^. Interestingly, *BCL2* is a target for one of the miRNAs that are downregulated in KS patients, thus it could be the testicular germ cell loss may be related to misregulation of *BCL2*.

Evidence of the function of these genes in spermatogenesis and development, as well as their participation in the differentiation of spermatogonia, may explain how alterations to the proliferation mechanisms of SSCs inhibit the maintenance and survival of undifferentiated spermatogonial cells and thus abrogate spermatogenesis in the long term. Our results are consistent with evidence showing their role in oligozoospermia in KS, but further studies are necessary to assess the role(s) of these miRNA-based mechanisms in the reproductive phenotype of Klinefelter patients.

This is the first report of a miRNA expression profile based on testicular tissue from patients with Klinefelter syndrome. We identified 166 miRNAs that were differentially expressed. Of these, 27 were particularly abundant: 20 downregulated and 7 upregulated in samples derived of the KS patients compared to normal controls. These miRNAs have target genes that are related to spermatogenesis, which could inform future approaches to the evaluation of the mechanisms involved in the infertility of Klinefelter syndrome patients.

## Materials and methods

### Study design

A descriptive, transversal, comparative, case–control study was designed to analyse the expression profile of miRNAs in the testicular tissue of patients with Klinefelter syndrome and to compare it to the profile obtained for the normal testicular tissue. This study was approved by the Ethics Committee of School of Medicine, Universidad Autónoma de Nuevo León. Informed consent was obtained for donating the tissues to the biobank, and all the samples and information accessed by the authors were fully anonymized. All methods were performed in accordance with the relevant guidelines and regulations.

### Sample collection

Paraffin-embedded samples of testicular tissue from KS patients (cases) and subjects with obstructive azoospermia (controls) were obtained from the Department of Pathology Anatomy of the University Hospital La Paz (Madrid, Spain). In general, a G band karyotype (50 metaphases) was obtained to confirm the characterizations of the KS and normal chromosome sets. Additionally, a histopathological examination was performed to analyse the presence or absence of germ cells.

Three KS patients had a karyotype formula of 47,XXY, and one patient had a mosaic formula (47,XXY/46,XY). Patient ages were in the range of 33–36 years, and control individuals in the 30–40-year range. For three (3) patients, two samples (one from each testis) were obtained and labelled as KLI_1 to KLI_6; the fourth patient had only one sample available (KLI_7). All the samples showed seminiferous tubules with spermatogenesis (1–5%), sclerosis (75–90%), and Sertoli cells-only (10–25%). Histological characteristics of the samples are detailed in Supplementary data Table S6.

All control samples were derived from biopsies of patients with infertility because of azoospermia with an age range of 30–40 years. In these samples, the seminiferous tubules had complete spermatogenesis with changes that suggested the presence of obstruction like tubular ectasia, seminiferous epithelium irregular in height, vacuolization of the apical pole of Sertoli cells and a higher number of adult spermatids than young spermatids per cross tubular section. These observations and the normal spermatogonia counts led to the diagnosis of obstructive azoospermia. No seminiferous tubules with Sertoli cells-only or with sclerosis were observed. All of the controls had a 46,XY karyotype. Each sample was processed and analysed separately (i.e., no pooled samples). For each sample, from five to ten paraffin 15-micron sections were cut with a microtome, placed in Eppendorf tubes, and labelled with a numeric key.

### Extraction of total RNA

Total RNA extraction was performed with a mirVana™ miRNA isolation kit (Life Technologies, Grand Island, NY) from the paraffin sections as instructed by the vendor. The purity of the RNA samples were measured with an Agilent 2,100 Bioanalyzer and the Agilent RNA 6,000 Pico Kit (Agilent, CA, USA). The samples used for analyses contained at least 50 nanograms of total RNA with, at least, a 1.8 absorbance ratio (260/280 nm).

### Sample analysis

#### Library construction and sequencing

Approximately 1 µg of total RNA was used to prepare a small RNA library according to the instructions in the TruSeq small RNA Sample preparation kit (Illumina, San Diego, USA). Single-end sequencing of 50 bp was performed on an Illumina HiSeq 2,500 (Illumina, San Diego, USA). The raw sequence data is available at https://www.ncbi.nlm.nih.gov/bioproject/ PRJNA640051, BioProject: PRJNA640051, ID: 640,051.

#### Bioinformatics analysis

Raw reads were processed with ACGT101-miR (LC Sciences, Houston, Texas, USA) to remove adapter dimers, junk, low complexity reads, common RNA families (rRNA, tRNA, snRNA, and snoRNA) and repeats. Subsequently, unique sequences with lengths of 18–26 nucleotides were mapped to specific-species precursors obtained from the miRBase 22.0 by a BLAST search performed to identify known miRNAs and novel 3p- and 5p-derived miRNAs. The remaining sequences were aligned against the miRbase (Release 21) (https://www.miRbase.org/) miRNA database, and perfectly matched sequences were considered to be conserved *Homo sapiens* miRNAs.

The normalization of the sequence counts in each sample (or data set) was achieved by dividing the counts by a library size parameter of the corresponding sample. The library size parameter was the median value of the ratio of the counts in a specific sample to those in a pseudo-reference sample. The count number in a pseudo-reference sample is the geometric mean of the counts across all samples. The expression level for each miRNA was categorized in 3 groups: high (a sequence count higher than the average copy number in the data set), medium (a sequence count higher than 10 and less than the average copy number in the data set), and low (a sequence count lower than 10).

### Statistical analysis

Differential expression of miRNAs as a function of normalized deep sequence counting was analysed through the selective use of Student’s t-tests. The significance threshold was set at < 0.05 for each test. Paired samples from 3 patients (KLI_1 to KLI_6) were used to increase reproducibility. Patient number 4 had only one biopsy sample, and thus, there was no paired sample available, and this single sample was included to increase the statistical strength of our study.

#### miRNAs—target prediction and interaction networks

We carried out two separate interactome analyses of differentially and highly expressed miRNAs, one for the upregulated and another for the downregulated using the miRNet database platform^19,20^. This database integrates the information from 11 different miRNA databases: TarBase, miRTarBase, miRecords, miRanda, miR2Disease, HMDD, PhenomiR, SM2miR, Pharmaco-miR, EpimiR, and StarBase. The miRNA target prediction was followed by an interactome network analysis and a GO term enrichment for terms related to sex differentiation, male sex reproduction, and cell differentiation and development. The miRNA-predicted target genes were visualized with Cytoscape software (https://cytoscape.org/). Cytoscape is an open source software package that allows for powerful visual mappings of the provided datasets.

The target genes obtained were analysed through a statistical analysis of the over-representation test results using the PANTHER platform^59^. For P-value calculation for the over-representation test data, the ‘expected’ value is the number of genes expected to be added to the test list for a particular PANTHER category (GO biological process), based on a predetermined reference list. The list of target genes for the differentially overexpressed miRNAs was used to calculate the p-value with Fisher test with a significance value of *p* < 0.05.

## Supplementary information

Supplementary information.

## Data Availability

The datasets generated and/or analysed during the current study are available in the Sequence Read Archive (SRA) repository, https://www.ncbi.nlm.nih.gov/bioproject/PRJNA640051, BioProject: PRJNA640051, ID: 640,051.
