# Supplementary material for: Expression profile of microRNAs in the testes of patients with Klinefelter syndrome
Source: Sci Rep. 2020 Jul 10;10:11470. doi: 10.1038/s41598-020-68294-7 (PMC7351945; doi:10.1038/s41598-020-68294-7)
Supplement: Supplementary file 1 — Supplementary information. [file 41598_2020_68294_MOESM1_ESM.pdf]

# EXPRESSION PROFILE OF microRNAs IN THE TESTES OF PATIENTS WITH KLINEFELTER SYNDROME

Ibarra-Ramírez Marisol[1], Calvo-Anguiano Geovana[1], Lugo-Trampe José de Jesús[1], Martínez-de-Villarreal Laura Elia[1], Rodríguez-Torres David[1], Nistal Manuel,[2] González-Peramato Pilar[3]

[1] Departamento de Genética, Facultad de Medicina y Hospital Universitario José E. González, Universidad Autónoma de Nuevo León (UANL), Av. Gonzalitos s/n cruce con Av. Madero, Col. Mitras Centro CP 64460, Monterrey, N.L., México

[2] Departamento de Anatomía, Histología y Neurociencias. Universidad Autónoma de Madrid. C/Arzobispo Morcillo 4 CP 28029 Madrid, España

[3] Departamento de Anatomía Patológica, Hospital Universitario La Paz y Universidad Autónoma de Madrid. C/Arzobispo Morcillo 4 CP 28029 Madrid, España

Corresponding author

**González-Peramato Pilar**

Departamento de Anatomía Patológica, Hospital Universitario La Paz y Universidad Autónoma de Madrid. C/Arzobispo Morcillo 4 CP 28029 Madrid, España

Tel: +34 914 97 50 00

Email: mpilar.gonzalezperamato@salud.madrid.org

**Table S1. List of differentially expressed miRNAs with p values <0.05.**

| miR_name | miR_seq | up/down | nge(Case(mean)/Control | log2(fold_change) | pvalue(t_test) | Control (mean) | Case(mean) | Case/KLI_1(norm) | Expression level |
|---|---|---|---|---|---|---|---|---|---|
| hsa-miR-106b-5p | TAAAGTGCTGACAGTGCAGAT | down | 0.43 | -1.23 | 7.75E-05 | 467 | 199 | 139 | high |
| hsa-miR-30a-3p | CTTTCAGTCGGATGTTTGCAGC | down | 0.38 | -1.39 | 9.56E-05 | 1,013 | 387 | 260 | high |
| hsa-miR-130a-3p | CAGTGCAATGTTAAAAGGGCAT | down | 0.43 | -1.22 | 2.91E-04 | 699 | 301 | 164 | middle |
| hsa-miR-16-5p | TAGCAGCACGTAAATATTGGCG | down | 0.41 | -1.29 | 3.62E-04 | 4,173 | 1,707 | 1,086 | high |
| hsa-miR-30a-5p | TGTAAACATCCTCGACTGGAAGCT | down | 0.38 | -1.39 | 9.21E-04 | 12,668 | 4,836 | 5,006 | high |
| hsa-miR-126-3p | TCGTACCGTGAGTAATAATGC | down | 0.52 | -0.93 | 1.22E-03 | 6,112 | 3,197 | 2,674 | high |
| hsa-miR-28-5p | AAGGAGCTCACAGTCTATTGA | down | 0.64 | -0.65 | 1.22E-03 | 264 | 168 | 126 | middle |
| hsa-miR-425-3p | CATCGGGAATGTCGTGTCCGCC | down | 0.42 | -1.26 | 1.24E-03 | 55 | 23 | 25 | middle |
| hsa-miR-425-5p | AATGACACGATCACTCCCGTTGAGT | down | 0.32 | -1.63 | 1.33E-03 | 683 | 221 | 223 | middle |
| hsa-miR-296-5p | AGGGCCCCCCCTCAATCCTGT | down | 0.44 | -1.20 | 1.36E-03 | 103 | 45 | 34 | middle |
| hsa-miR-31-3p | TGCTATGCCAACATATTGCCATC | down | 0.29 | -1.77 | 1.45E-03 | 37 | 11 | 7 | middle |
| hsa-miR-17-5p | CAAAGTGCTTACAGTGCAGGTAG | down | 0.27 | -1.90 | 1.58E-03 | 845 | 227 | 151 | high |
| hsa-miR-106a-5p | CAAAGTGCTTACAGTGCAGGTAG | down | 0.27 | -1.90 | 1.58E-03 | 845 | 227 | 151 | high |
| hsa-miR-16-1-3p | CCAGTATTAACTGTGCTGCTGA | down | 0.22 | -2.21 | 1.62E-03 | 23 | 5 | 5 | middle |
| hsa-miR-203b-3p | TTGAACTGTCAAGAACCACTGG | down | 0.09 | -3.51 | 2.79E-03 | 5 | 0 | 0 | low |
| hsa-miR-3180-5p | CTTCCAGACGCTCCGCCCCACT | down | 0.04 | -4.66 | 2.86E-03 | 5 | 0 | 0 | low |
| hsa-miR-181c-5p | AACATTCAACCTGTCGGTGAGT | down | 0.51 | -0.98 | 3.20E-03 | 147 | 75 | 33 | middle |
| hsa-miR-592 | TTGTGTCAATATGCGATGATGT | down | 0.03 | -4.89 | 3.22E-03 | 7 | 0 | 0 | middle |
| hsa-miR-935 | CAGTTACCGCTTCCGCTACCGC | down | 0.15 | -2.75 | 3.30E-03 | 26 | 4 | 4 | middle |
| hsa-miR-525-3p | GAAGGCGCTTCCCTTTAGAGC | down | -inf | -inf | 3.97E-03 | 2 | 0 | 0 | low |
| hsa-miR-629-3p | GTTCTCCCAACGTAAGCCCAGC | down | 0.05 | -4.27 | 4.15E-03 | 5 | 0 | 0 | low |
| hsa-miR-130b-5p | ACTCTTTCCCTGTTGCACTACT | down | 0.31 | -1.69 | 4.43E-03 | 97 | 30 | 43 | middle |
| hsa-miR-449b-3p | CAGCCACAACTACCCTGCCACT | down | 0.01 | -7.22 | 5.02E-03 | 65 | 0 | 1 | middle |
| hsa-miR-25-3p | CATTGCACTTGTCTCGGTCTGA | down | 0.45 | -1.16 | 5.13E-03 | 3,820 | 1,707 | 1,582 | high |
| hsa-miR-30c-5p | TGTAAACATCCTACACTCTCAGCT | down | 0.51 | -0.97 | 5.49E-03 | 9,447 | 4,815 | 4,595 | high |
| hsa-miR-519a-2-5p | CCTCTACAGGGAAGCGCTTTCT | down | 0.03 | -4.94 | 5.84E-03 | 6 | 0 | 0 | low |
| hsa-miR-7162-3p | TCTGAGGTGGAACAGCAGC | down | -inf | -inf | 6.02E-03 | 7 | 0 | 0 | low |
| hsa-miR-17-3p | ACTGCAGTGAGGGCACTTGTAG | down | 0.43 | -1.21 | 6.38E-03 | 163 | 70 | 36 | middle |
| hsa-miR-20b-5p | CAAAGTGCTCATAGTGCAGGTAG | down | 0.16 | -2.65 | 6.38E-03 | 60 | 10 | 5 | middle |
| hsa-miR-27a-5p | AGGGCTTAGCTGCTTGTGAGCA | down | 0.55 | -0.87 | 7.09E-03 | 28 | 16 | 15 | middle |
| hsa-miR-891a-5p | TGCAACGAACCTGAGCCACTGA | down | 0.02 | -5.79 | 7.10E-03 | 83 | 1 | 0 | middle |
| hsa-miR-3940-3p | CAGCCCGGATCCCAGCCCACT | down | 0.20 | -2.31 | 7.73E-03 | 15 | 3 | 6 | middle |
| hsa-miR-375-3p | TTTGTTCGTTCGGCTCGCGTGA | down | 0.29 | -1.76 | 7.73E-03 | 160 | 47 | 38 | middle |
| hsa-let-7g-5p | TGAGGTAGTAGTTTGTACAGTT | down | 0.61 | -0.71 | 8.96E-03 | 11,277 | 6,910 | 4,759 | high |
| hsa-miR-92a-3p | TATTGCACTTGTCCCGGCCTGT | down | 0.65 | -0.62 | 1.01E-02 | 11,412 | 7,444 | 8,112 | high |
| hsa-miR-767-3p | TCTGCTCATACCCCATGGTTTC | down | 0.03 | -5.08 | 1.13E-02 | 4 | 0 | 0 | low |
| hsa-miR-182-5p | TTTGGCAATGGTAGAACTCACACT | down | 0.45 | -1.14 | 1.20E-02 | 392 | 178 | 175 | middle |
| hsa-miR-18a-3p | ACTGCCCTAAGTGCTCCTTCTGT | down | 0.17 | -2.54 | 1.26E-02 | 42 | 7 | 7 | middle |
| hsa-miR-15b-5p | TAGCAGCACATCATGGTTTACA | down | 0.34 | -1.56 | 1.29E-02 | 1,715 | 580 | 517 | high |
| hsa-miR-216b-5p | AAATCTCTGCAGGCAAATGTGA | down | 0.03 | -5.00 | 1.29E-02 | 12 | 0 | 0 | middle |
| hsa-miR-7-1-3p | CAACAAATCACAGTCTGCCATT | down | 0.18 | -2.49 | 1.33E-02 | 88 | 16 | 5 | middle |
| hsa-miR-18b-5p | TAAGGTGCATCTAGTGCAGTT | down | 0.09 | -3.55 | 1.50E-02 | 5 | 0 | 1 | low |
| hsa-miR-518d-3p | CAAAGCGCTTCCCTTTGGAGC | down | -inf | -inf | 1.67E-02 | 3 | 0 | 0 | low |
| hsa-miR-371a-5p | ACTCAAACTGTGGGGGCACTTT | down | 0.09 | -3.49 | 1.80E-02 | 12 | 1 | 0 | middle |
| hsa-miR-767-5p | TGCACCATGGTTGTCTGAGCATGC | down | -inf | -inf | 1.85E-02 | 8 | 0 | 0 | middle |
| hsa-miR-103a-3p | AGCAGCATTGTACAGGGCTATGA | down | 0.67 | -0.58 | 1.90E-02 | 1,666 | 1,115 | 826 | high |
| hsa-miR-520a-5p | CTCCAGAGGGAAGTACTTTCT | down | 0.06 | -4.18 | 2.06E-02 | 31 | 2 | 2 | middle |
| hsa-miR-105-5p | TCAAATGCTCAGACTCCTGTGGT | down | 0.03 | -5.21 | 2.08E-02 | 6 | 0 | 0 | middle |
| hsa-miR-10a-5p | TACCCTGTAGATCCGAATTTGT | down | 0.30 | -1.75 | 2.22E-02 | 6,198 | 1,842 | 1,787 | high |
| hsa-miR-518b | CAAAGCGCTCCCCTTTAGAGGT | down | 0.05 | -4.42 | 2.26E-02 | 51 | 2 | 6 | middle |
| hsa-miR-4433b-5p | ATGTCCCACCCCCACTCCTGTT | down | 0.23 | -2.13 | 2.30E-02 | 85 | 19 | 7 | middle |
| hsa-miR-520h | ACAAAGTGCTTCCCTTTAGAGT | down | 0.05 | -4.23 | 2.37E-02 | 9 | 0 | 1 | middle |

| | | | | | | | | | |
|---|---|---|---|---|---|---|---|---|---|
| hsa-mir-12127-p3 | TAAAGGTATACTCGTTACAGGCCC | down | 0.06 | -4.07 | 2.51E-02 | 3 | 0 | 1 | low |
| hsa-miR-519d-3p | CAAAGTGCCTCCCTTTAGAGTGT | down | 0.02 | -5.40 | 2.56E-02 | 70 | 2 | 1 | middle |
| hsa-miR-92a-1-5p | AGGTTGGGATCGGTTGCAATGCT | down | 0.33 | -1.60 | 2.62E-02 | 12 | 4 | 5 | middle |
| hsa-miR-525-5p_R+1 | CTCCAGAGGGATGCACTTTCTC | down | 0.22 | -2.18 | 2.63E-02 | 15 | 3 | 11 | middle |
| hsa-miR-34c-3p | AATCACTAACCACACGGCCAGG | down | 0.10 | -3.30 | 2.69E-02 | 170 | 17 | 4 | middle |
| hsa-miR-142-3p | GTAGTGTTTCCTACTTTATGGA | down | 0.34 | -1.57 | 2.74E-02 | 2,093 | 707 | 278 | high |
| hsa-miR-532-5p | CATGCCTTGAGTGTAGGACCGT | down | 0.82 | -0.29 | 2.76E-02 | 489 | 399 | 309 | middle |
| hsa-miR-101-2-5p | TCGGTTATCATGGTACCGATGCT | down | 0.23 | -2.13 | 2.76E-02 | 2 | 1 | 1 | low |
| hsa-miR-760 | CGGCTCTGGGTCTGTGGGGAGT | down | 0.29 | -1.81 | 2.78E-02 | 19 | 6 | 5 | middle |
| hsa-miR-21-5p | TAGCTTATCAGACTGATGTTGAC | down | 0.52 | -0.96 | 2.78E-02 | 51,252 | 26,412 | 17,505 | high |
| hsa-miR-23b-3p | ATCACATTGCCAGGGATTACC | down | 0.50 | -0.99 | 2.85E-02 | 3,515 | 1,769 | 1,117 | high |
| hsa-miR-625-3p | GACTATAGAACTTTCCCCCTCA | down | 0.16 | -2.67 | 2.98E-02 | 216 | 34 | 81 | middle |
| hsa-miR-421 | ATCAACAGACATTAATTGGGCGC | down | 0.65 | -0.61 | 3.01E-02 | 70 | 46 | 31 | middle |
| hsa-miR-518e-5p | CTCTAGAGGGAAGCGCTTTCT | down | 0.04 | -4.73 | 3.16E-02 | 10 | 0 | 1 | middle |
| hsa-miR-520f-5p | CTCTAGAGGGAAGCGCTTTCT | down | 0.04 | -4.73 | 3.16E-02 | 10 | 0 | 1 | middle |
| hsa-miR-34b-3p | AATCACTAACTCCACTGCCATC | down | 0.09 | -3.55 | 3.17E-02 | 431 | 37 | 21 | middle |
| hsa-miR-15b-3p | CGAATCATTATTTGCTGCTCT | down | 0.34 | -1.54 | 3.38E-02 | 148 | 51 | 45 | middle |
| hsa-miR-148b-3p | TCAGTGCATCACAGAACTTTGT | down | 0.65 | -0.63 | 3.44E-02 | 1,233 | 797 | 614 | high |
| hsa-miR-518e-3p | AAAGCGCTTCCCTTCAGAGTGT | down | 0.02 | -5.48 | 3.44E-02 | 29 | 1 | 1 | middle |
| hsa-miR-192-3p | CTGCCAATTCCATAGGTCACAGT | down | 0.05 | -4.31 | 3.54E-02 | 55 | 3 | 2 | middle |
| hsa-miR-363-3p | AATTGCACGGTATCCATCTGTA | down | 0.40 | -1.31 | 3.60E-02 | 150 | 60 | 67 | middle |
| hsa-miR-3200-3p | CACCTTGCGCTACTCAGGTCTGT | down | 0.16 | -2.69 | 3.61E-02 | 15 | 2 | 2 | middle |
| hsa-miR-215-5p | ATGACCTATGAATTGACAGACA | down | 0.47 | -1.09 | 3.73E-02 | 37 | 17 | 25 | middle |
| hsa-miR-4755-5p | TTTCCCTTCAGAGCCTGGCTTA | down | -inf | -inf | 3.74E-02 | 1 | 0 | 0 | low |
| hsa-mir-518b-p5 | CTCCAGAGGGAAGCGCTTTCT | down | 0.08 | -3.69 | 3.76E-02 | 2 | 0 | 0 | low |
| hsa-miR-93-5p | CAAAGTGCTGTTCGTGCAGGTAG | down | 0.29 | -1.80 | 3.96E-02 | 1,657 | 477 | 452 | high |
| hsa-miR-340-5p | TTATAAAGCAATGAGACTGATT | down | 0.40 | -1.34 | 3.96E-02 | 544 | 215 | 206 | middle |
| hsa-miR-3614-3p | TAGCCTTCAGATCTTGGTGTTT | down | 0.11 | -3.24 | 3.96E-02 | 3 | 0 | 2 | low |
| hsa-miR-892a | CACTGTGTCCTTTCTGCGTAGA | down | -inf | -inf | 3.98E-02 | 3 | 0 | 0 | low |
| hsa-miR-181a-5p | AACATTCAACGCTGTCGGTGAGT | down | 0.58 | -0.78 | 4.01E-02 | 1,315 | 768 | 769 | high |
| hsa-miR-18b-3p | TACTGCCCTAAATGCCCCTTCT | down | 0.07 | -3.91 | 4.09E-02 | 3 | 0 | 0 | low |
| hsa-miR-3158-3p | AAGGGCTTCCTCTCTGCAGGAC | down | 0.04 | -4.79 | 4.15E-02 | 10 | 0 | 0 | middle |
| hsa-miR-371a-3p | AAGTGCCGCCATCTTTTGAGTGT | down | -inf | -inf | 4.18E-02 | 5 | 0 | 0 | low |
| hsa-miR-518a-3p | AAAGCGCTTCCCTTTGCTGGAT | down | 0.01 | -6.89 | 4.23E-02 | 7 | 0 | 0 | middle |
| hsa-miR-3617-3p | CATCAGCACCCTATGTCCTTTC | down | -inf | -inf | 4.39E-02 | 1 | 0 | 0 | low |
| hsa-miR-340-3p | TCCGTCTCAGTTACTTTATAGC | down | 0.47 | -1.09 | 4.42E-02 | 104 | 49 | 27 | middle |
| hsa-miR-148a-5p | AAAGTTCTGAGACACTCCGACT | down | 0.56 | -0.83 | 4.44E-02 | 69 | 39 | 48 | middle |
| hsa-miR-498-3p | AAAGCACCTCCAGAGCTTGAAGC | down | -inf | -inf | 4.47E-02 | 12 | 0 | 0 | middle |
| hsa-miR-517-5p | CCTCTAGATGGAAGCACTGTCT | down | 0.10 | -3.31 | 4.49E-02 | 1 | 0 | 0 | low |
| hsa-miR-670-5p | GTCCCTGAGTGTATGTGGTGAAC | down | 0.21 | -2.27 | 4.59E-02 | 9 | 2 | 0 | middle |
| hsa-miR-7154-5p | TTCATGAACTGGGTCTAGCTTGGAGC | down | -inf | -inf | 4.74E-02 | 23 | 0 | 0 | middle |
| hsa-miR-128-3p | TCACAGTGAACCGGTCTCTTT | down | 0.57 | -0.82 | 4.78E-02 | 296 | 167 | 126 | middle |
| hsa-miR-515-3p | GAGTGCCTTCTTTTGGAGCGTT | down | -inf | -inf | 4.80E-02 | 7 | 0 | 0 | middle |
| hsa-miR-18a-5p | TAAGGTGCATCTAGTGCAGATAG | down | 0.30 | -1.76 | 4.83E-02 | 153 | 45 | 23 | middle |
| hsa-miR-212-5p | ACCTTGGCTCTAGACTGCTTACT | down | 0.67 | -0.58 | 4.86E-02 | 26 | 17 | 21 | middle |
| hsa-miR-518d-5p | CTCTAGAGGGAAGCACTTTCT | down | 0.02 | -5.87 | 4.86E-02 | 10 | 0 | 0 | middle |
| hsa-miR-518f-5p | CTCTAGAGGGAAGCACTTTCT | down | 0.02 | -5.87 | 4.86E-02 | 10 | 0 | 0 | middle |
| hsa-miR-4780 | ACCCTTGAGCCTGATCCCTAGC | down | 0.11 | -3.20 | 4.93E-02 | 2 | 0 | 0 | low |
| hsa-miR-1185-1-3p | ATATACAGGGGGAGACTCTTAT | up | 4.25 | 2.09 | 9.53E-04 | 6 | 26 | 27 | middle |
| hsa-miR-432-5p | TCTTGGAGTAGGTCATTGGGTGT | up | 4.08 | 2.03 | 1.18E-03 | 31 | 128 | 85 | middle |
| hsa-let-7b-3p | CTATACAACCTACTGCCTTCCT | up | 2.02 | 1.01 | 1.28E-03 | 401 | 807 | 854 | middle |
| hsa-miR-487b-3p | AATCGTACAGGGTCATCCACTT | up | 3.04 | 1.61 | 1.48E-03 | 72 | 218 | 133 | middle |
| hsa-miR-494-5p | AGGTTGTCCGTGTTGTCTTCT | up | 4.87 | 2.28 | 1.50E-03 | 1 | 6 | 5 | low |

| | | | | | | | | | |
|---|---|---|---|---|---|---|---|---|---|
| hsa-miR-7704 | CGGGGTCGGCGGCGACGTC | up | 9.79 | 3.29 | 2.16E-03 | 7 | 67 | 89 | middle |
| hsa-miR-433-3p | ATCATGATGGGCTCCTCGGTGT | up | 4.04 | 2.02 | 2.18E-03 | 15 | 61 | 76 | middle |
| hsa-miR-127-3p | TCGGATCCGTCTGAGCTTGGCT | up | 6.37 | 2.67 | 2.18E-03 | 1,802 | 11,477 | 14,269 | high |
| hsa-miR-379-5p | TGGTAGACTATGGAACGTAGG | up | 2.59 | 1.37 | 2.92E-03 | 120 | 310 | 211 | middle |
| hsa-miR-214-5p | TGCCTGTCTACACTTGCTGTGC | up | 2.60 | 1.38 | 3.17E-03 | 128 | 333 | 243 | middle |
| hsa-miR-134-5p | TGTGACTGGTTGACCAGAGGGG | up | 3.92 | 1.97 | 3.26E-03 | 112 | 438 | 439 | middle |
| hsa-miR-409-3p | GAATGTTGCTCGGTGAACCCCT | up | 2.72 | 1.44 | 3.30E-03 | 317 | 862 | 836 | middle |
| hsa-miR-431-5p | TGTCTTGCAGGCCGTCATGCA | up | 2.30 | 1.20 | 3.72E-03 | 8 | 19 | 15 | middle |
| hsa-miR-382-5p | GAAGTTGTTCGTGGTGGATTCG | up | 3.32 | 1.73 | 3.88E-03 | 14 | 47 | 38 | middle |
| hsa-miR-369-5p | AGATCGACCGTGTTATATTCG | up | 2.24 | 1.16 | 4.26E-03 | 26 | 58 | 40 | middle |
| hsa-miR-409-5p | AGGTTACCCGAGCAACTTTGCAT | up | 2.87 | 1.52 | 4.34E-03 | 212 | 610 | 712 | middle |
| hsa-miR-615-3p | TCCGAGCCTGGGTCTCCCTCT | up | 2.53 | 1.34 | 4.83E-03 | 26 | 67 | 83 | middle |
| hsa-miR-543 | AAACATTCGCGGTGCACTTCTT | up | 2.53 | 1.34 | 5.65E-03 | 33 | 83 | 76 | middle |
| hsa-miR-1291 | GTGGCCCTGACTGAAGACCAGC | up | 2.05 | 1.03 | 7.10E-03 | 61 | 125 | 191 | middle |
| hsa-miR-483-3p | CACTCCTCTCCTCCCGTCTTCT | up | 2.57 | 1.36 | 7.32E-03 | 123 | 316 | 291 | middle |
| hsa-miR-370-3p | GCCTGCTGGGGTGGAACCTGGT | up | 4.18 | 2.07 | 8.02E-03 | 34 | 140 | 227 | middle |
| hsa-let-7b-5p | TGAGGTAGTAGGTTGTGTGGTT | up | 1.72 | 0.78 | 8.25E-03 | 8,520 | 14,620 | 15,054 | high |
| hsa-miR-323a-3p | GCACATTACACGGTCGACCTCT | up | 4.48 | 2.16 | 8.57E-03 | 30 | 133 | 102 | middle |
| hsa-miR-708-5p | AAGGAGCTTACAATCTAGCTGG | up | 2.67 | 1.42 | 9.50E-03 | 100 | 269 | 198 | middle |
| hsa-miR-675-3p | CTGTATGCCCTCACCGCTCAGC | up | 2.70 | 1.43 | 9.57E-03 | 33 | 89 | 80 | middle |
| hsa-miR-493-5p | TTGTACATGGTAGGCTTTCATT | up | 2.93 | 1.55 | 1.02E-02 | 108 | 317 | 194 | middle |
| hsa-miR-329-3p | AACACACCTGGTTAACCTCTTT | up | 3.28 | 1.71 | 1.09E-02 | 13 | 43 | 54 | middle |
| hsa-miR-4485-3p | CGGCCGCGGTACCCTAAC | up | 6.21 | 2.63 | 1.11E-02 | 15 | 96 | 126 | middle |
| hsa-miR-323b-3p | CCCAATACACGGTCGACCTCT | up | 5.27 | 2.40 | 1.15E-02 | 17 | 87 | 92 | middle |
| hsa-miR-125b-2-3p | ACAAGTCAGGCTCTTGGGACCT | up | 1.88 | 0.91 | 1.17E-02 | 257 | 484 | 512 | middle |
| hsa-miR-381-3p | TATACAAGGGCAAGCTCTCTGT | up | 2.84 | 1.51 | 1.18E-02 | 204 | 581 | 498 | middle |
| hsa-miR-199a-5p | CCCAGTGTTCAGACTACCTGTTC | up | 2.54 | 1.35 | 1.37E-02 | 8,901 | 22,651 | 24,542 | high |
| hsa-miR-377-5p | AGAGGTTGCCCTTGGTGAATTC | up | 4.55 | 2.19 | 1.40E-02 | 13 | 60 | 61 | middle |
| hsa-miR-1247-5p | ACCCGTCCCGTTCGTCCCCGGA | up | 6.53 | 2.71 | 1.49E-02 | 83 | 541 | 654 | middle |
| hsa-miR-381-5p | AGCGAGGTTGCCCTTTGTATATT | up | 5.00 | 2.32 | 1.49E-02 | 2 | 12 | 11 | middle |
| hsa-miR-299-3p | TATGTGGGATGGTAAACCGCTT | up | 2.34 | 1.22 | 1.50E-02 | 47 | 109 | 48 | middle |
| hsa-miR-485-5p | AGAGGCTGGCCGTGATGAATTCG | up | 4.62 | 2.21 | 1.57E-02 | 9 | 42 | 4 | middle |
| hsa-miR-193b-3p | AACTGGCCCTCAAAGTCCCGCT | up | 2.00 | 1.00 | 1.61E-02 | 459 | 919 | 781 | middle |
| hsa-miR-1251-5p | ACTCTAGCTGCCAAAGGCGCT | up | 24.99 | 4.64 | 1.68E-02 | 0 | 7 | 13 | middle |
| hsa-miR-125a-5p | TCCCTGAGACCCTTTAACCTGT | up | 2.41 | 1.27 | 1.76E-02 | 23,767 | 57,221 | 74,003 | high |
| hsa-miR-125b-5p | TCCCTGAGACCCTAACTTGTGA | up | 1.97 | 0.98 | 1.79E-02 | 20,416 | 40,270 | 48,785 | high |
| hsa-mir-10401-p5 | AGGCGTGGGGTGCGGACCC | up | 4.02 | 2.01 | 1.81E-02 | 2 | 8 | 6 | middle |
| hsa-miR-1468-5p | CTCCGTTTGCCTGTTTCGCTGA | up | 5.32 | 2.41 | 1.87E-02 | 16 | 86 | 86 | middle |
| hsa-miR-214-3p | ACAGCAGGCACAGACAGGCAGT | up | 2.03 | 1.02 | 2.06E-02 | 374 | 761 | 624 | middle |
| hsa-miR-708-3p | CAACTAGACTGTGAGCTTCTAG | up | 2.41 | 1.27 | 2.08E-02 | 13 | 32 | 29 | middle |
| hsa-miR-4508 | AGCGGGGCTGGGCGCGCGC | up | 13.92 | 3.80 | 2.10E-02 | 3 | 37 | 83 | middle |
| hsa-miR-654-5p | TGGTGGGCCGCAGAACATGTGC | up | 4.54 | 2.18 | 2.19E-02 | 5 | 23 | 28 | middle |
| hsa-miR-665 | ACCAGGAGGCTGAGGCCCCT | up | 5.20 | 2.38 | 2.29E-02 | 2 | 12 | 6 | middle |
| hsa-miR-134-3p | CTGTGGGCCACCTAGTCACCA | up | 6.28 | 2.65 | 2.64E-02 | 2 | 16 | 11 | middle |
| hsa-miR-1197 | TAGGACACATGGTCTACTTCT | up | 3.97 | 1.99 | 2.84E-02 | 2 | 9 | 13 | middle |
| hsa-miR-1299 | TTCTGGAATTCTGTGTGAGGGA | up | 7.48 | 2.90 | 3.16E-02 | 4 | 28 | 60 | middle |
| hsa-miR-410-3p | AATATAACACAGATGGCCTGT | up | 2.24 | 1.16 | 3.26E-02 | 47 | 104 | 70 | middle |
| hsa-miR-127-5p | CTGAAGCTCAGAGGGCTCTGATT | up | 2.49 | 1.32 | 3.39E-02 | 83 | 207 | 112 | middle |
| hsa-miR-496 | AGTATTACATGGCCAATCTCC | up | 2.33 | 1.22 | 3.47E-02 | 7 | 16 | 4 | middle |
| hsa-miR-1224-3p | CCCCACCTCCTCTCTCCTCAGT | up | 3.51 | 1.81 | 3.57E-02 | 4 | 12 | 6 | middle |
| hsa-miR-4492 | CGGGGCTGGGCGCGCGCC | up | 23.12 | 4.53 | 3.68E-02 | 2 | 52 | 52 | middle |
| hsa-miR-199b-3p | ACAGTAGTCTGCACATTGGTT | up | 2.05 | 1.03 | 3.78E-02 | 2,737 | 5,604 | 4,460 | high |
| hsa-miR-889-3p | TTAATATCGGACAACCATTGT | up | 2.35 | 1.23 | 4.11E-02 | 56 | 131 | 135 | middle |
| hsa-miR-485-3p | GTCATACACGGCTCTCCTCTCT | up | 3.12 | 1.64 | 4.27E-02 | 39 | 123 | 148 | middle |
| hsa-mir-7108-p3 | CCCCGCCCCACCCCGCGC | up | 7.80 | 2.96 | 4.56E-02 | 3 | 25 | 33 | middle |
| hsa-miR-1908-5p | CGGCGGGGACGGCGATTGGT | up | 24.90 | 4.64 | 4.73E-02 | 0 | 4 | 7 | low |
| hsa-miR-125b-1-3p | ACGGGTTAGGCTCTTGGGAGCT | up | 1.49 | 0.57 | 4.83E-02 | 64 | 95 | 95 | middle |
| hsa-miR-337-5p | GAACGGCTTCATACAGGAGT | up | 2.16 | 1.11 | 4.87E-02 | 18 | 38 | 29 | middle |
| hsa-miR-323a-5p | AGGTGGTCCGTGGCGCGTTCGC | up | inf | inf | 4.92E-02 | 0 | 1 | 1 | low |
| | | | | | | | | | |

**Table S1.** List of differentially expressed hsa-miRNAs with p values <0.05.

| | | hsa-miR-125b-5p | hsa-miR-125a-5p | hsa-let-7b-5p | hsa-miR-199a-5p | hsa-miR-199b-3p | hsa-miR-654-3p | hsa-miR-127-3p |
|---|---|---|---|---|---|---|---|---|
| *ABCC1* | ATP-binding cassette, sub-family C (CFTR/MRP), member 1 | + | | + | + | | | |
| *ABL1* | c-abl oncogene 1, non-receptor tyrosine kinase | + | | + | | | | |
| *ACTN4* | actinin alpha 4 | | | + | | | + | |
| *AKT1* | thymoma viral proto-oncogene 1 | + | + | | | | + | |
| *ARID3A* | AT rich interactive domain 3A (BRIGHT-like) | + | + | + | | | | |
| *ARID3B* | AT rich interactive domain 3B (BRIGHT-like) | + | + | + | | | | |
| *BTG2* | BTG anti-proliferation factor 2 | + | + | + | | | | |
| *CDKN1A* | cyclin-dependent kinase inhibitor 1A (P21) | | + | + | | | + | |
| *CLDN12* | claudin 12 | + | + | + | | | | |
| *CSNK2A1* | casein kinase 2, alpha 1 polypeptide | + | + | + | + | | | |
| *DHX33* | DEAH (Asp-Glu-Ala-His) box polypeptide 33 | + | + | + | | | | |
| *E2F2* | E2F transcription factor 2 | + | | + | | + | | |
| *E2F3* | E2F transcription factor 3 | + | + | + | | | | |
| *E2F7* | E2F transcription factor 7 | + | + | + | | | | |
| *EDN1* | endothelin 1 | | + | + | + | | | |
| *EEF1A1* | eukaryotic translation elongation factor 1 alpha 1 | + | + | + | | | | |
| *ERBB2* | erb-b2 receptor tyrosine kinase 2 | + | + | | + | | | |
| *ERBB3* | erb-b2 receptor tyrosine kinase 3 | + | + | | + | | | |
| *ETS1* | E26 avian leukemia oncogene 1, 5' domain | + | | | + | | + | |
| *EZH2* | enhancer of zeste 2 polycomb repressive complex 2 subunit | | | + | + | | | |
| *GPAT4* | glycerol-3-phosphate acyltransferase 4 | + | + | + | | | | |
| *HK2* | hexokinase 2 | + | + | | + | | | |
| *IP6K1* | inositol hexaphosphate kinase 1 | + | + | + | | | | |
| *ITGA3* | integrin alpha 3 | | | + | + | + | | |
| *JAG1* | jagged 1 | | | + | + | + | | |
| *LIF* | leukemia inhibitory factor | + | + | | + | | | |
| *LIN28A* | lin-28 homolog A (C. elegans) | + | + | + | | | | |
| *LIN28B* | lin-28 homolog B (C. elegans) | + | + | + | | | | |
| *OPRL1* | opioid receptor-like 1 | + | + | + | | | | |
| *PFKM* | phosphofructokinase, muscle | + | + | + | | | | |
| *PLXND1* | plexin D1 | + | | + | + | | | |
| *PRDM1* | PR domain containing 1, with ZNF domain | + | + | + | | | | + |
| *SEMA4C* | sema domain, immunoglobulin domain (Ig), transmembrane dor | + | + | + | | | | |
| *SGPL1* | sphingosine phosphate lyase 1 | + | + | | | | | |
| *SMAD4* | SMAD family member 4 | + | + | | + | | | |
| *TNFRSF10B* | tumor necrosis factor receptor superfamily, member 10b | + | + | + | | | | |
| *VPS51* | VPS51 GARP complex subunit | + | + | + | | | | |
| *WNK1* | WNK lysine deficient protein kinase 1 | + | | + | + | | | |
| *YOD1* | YOD1 deubiquitinase | + | + | + | | | | |

**Table S2. List of target genes for the 7 high-expression, differentially expressed miRNAs.** The list shows a select group of 39 which according to their GO terms could be associated to alterations in the spermatogenesis in Klinefelter Syndrome. List of 7 miRNAs upregulated that showed the highest expression levels and their interactions with target genes.

| Input | Name |
|---|---|
| *ABCB7* | ATP-binding cassette, sub-family B (MDR/TAP), member 7 |
| *ABCF1* | ATP-binding cassette, sub-family F (GCN20), member 1 |
| *ABL2* | v-abl Abelson murine leukemia viral oncogene 2 (arg, Abelson-related gene) |
| *ACOX1* | acyl-Coenzyme A oxidase 1, palmitoyl |
| *ACP2* | acid phosphatase 2, lysosomal |
| *ACVR2A* | activin receptor IIA |
| *AK2* | adenylate kinase 2 |
| *AP2A1* | adaptor-related protein complex 2, alpha 1 subunit |
| *AP2B1* | adaptor-related protein complex 2, beta 1 subunit |
| *ARCN1* | archain 1 |
| *ASGR2* | asialoglycoprotein receptor 2 |
| *ATOX1* | antioxidant 1 copper chaperone |
| *ATP5A1* | ATP synthase, H+ transporting, mitochondrial F1 complex, alpha subunit 1 |
| *BCL2* | B cell leukemia/lymphoma 2 |
| *BDNF* | brain derived neurotrophic factor |
| *BIRC5* | baculoviral IAP repeat-containing 5 |
| *BMI1* | Bmi1 polycomb ring finger oncogene |
| *CALU* | calumenin |
| *CAPZA2* | capping protein (actin filament) muscle Z-line, alpha 2 |
| *CCND1* | cyclin-dependent kinase inhibitor 1A (P21) |
| *CCNE2* | cyclin E2 |
| *CCNT2* | cyclin T2 |
| *CDC20* | cell division cycle 20 |
| *CDC25A* | cell division cycle 25A |
| *CDK6* | cyclin-dependent kinase 6 |
| *CDKN1A* | cyclin-dependent kinase inhibitor 1A (P21) |
| *CFL2* | cofilin 2, muscle |
| *COL4A2* | collagen, type IV, alpha 2 |
| *CRK* | v-crk avian sarcoma virus CT10 oncogene homolog |
| *CRKL* | v-crk avian sarcoma virus CT10 oncogene homolog-like |
| *DDX3X* | DEAD box helicase 3, X-linked |
| *DDX6* | DEAD (Asp-Glu-Ala-Asp) box polypeptide 6 |
| *DNMT1* | DNA methyltransferase (cytosine-5) 1 |
| *E2F3* | E2F transcription factor 3 |
| *EEF1A1* | eukaryotic translation elongation factor 1 alpha 1 |
| *EGFR* | epidermal growth factor receptor |
| *EIF5* | eukaryotic translation initiation factor 5 |

| | |
|---|---|
| *EN2* | engrailed 2 |
| *ENTPD1* | ectonucleoside triphosphate diphosphohydrolase 1 |
| *FGF2* | fibroblast growth factor 2 |
| *FURIN* | furin (paired basic amino acid cleaving enzyme) |
| *GPRC5A* | G protein-coupled receptor, family C, group 5, member A |
| *H3F3B* | H3.3 histone B |
| *HMGA2* | high mobility group AT-hook 2 |
| *HOXA3* | homeobox A3 |
| *HSP90AA1* | heat shock protein 90, alpha (cytosolic), class A member 1 |
| *HSPA1B* | heat shock protein 1B |
| *HSPA8* | heat shock protein 8 |
| *ITGA2* | integrin alpha 2 |
| *KCND3* | potassium voltage-gated channel, Shal-related family, member 3 |
| *KMT2A* | lysine (K)-specific methyltransferase 2A |
| *KPNA1* | karyopherin (importin) alpha 1 |
| *KPNA2* | karyopherin (importin) alpha 2 |
| *KRAS* | Kirsten rat sarcoma viral oncogene homolog |
| *MCL1* | myeloid cell leukemia sequence 1 |
| *MORF4L1* | mortality factor 4 like 1 |
| *MYC* | myelocytomatosis oncogene |
| *NOTCH2* | notch 2 |
| *NR2C2* | nuclear receptor subfamily 2, group C, member 2 |
| *PRKAA1* | protein kinase, AMP-activated, alpha 1 catalytic subunit |
| *SLC11A2* | solute carrier family 11 (proton-coupled divalent metal ion transporters), member 2 |
| *SLC7A5* | solute carrier family 7 (cationic amino acid transporter, y+ system), member 5 |
| *STAU1* | staufen double-stranded RNA binding protein 1 |
| *SZRD1* | SUZ RNA binding domain containing 1 |
| *TNPO1* | transportin 1 |
| *TNRC6B* | trinucleotide repeat containing 6b |
| *TWF1* | twinfilin actin binding protein 1 |
| *TXNIP* | thioredoxin interacting protein |
| *WEE1* | WEE 1 homolog 1 (S. pombe) |
| *ZFHX3* | zinc finger homeobox 3 |

**Table S3. List of target genes for the 20 downregulated with high-expression, differentially expressed miRNAs.** The list shows a select group of 70 which according to their GO terms could be associated to alterations in the spermatogenesis in Klinefelter Syndrome.

| GO biological process complete | Homo sapiens - REF LIST | miRNAs Target Input | miRNAs Target Input (expected) | miRNAs Target Input (fold Enrichment) | miRNAs Target Input (raw P-value) |
|---|---|---|---|---|---|
| Cell Differentiation (GO:0030154) | 3703 | 21 | 6.88 | 3.05 | 3.55E-07 |
| Stem Cell Differentiation (GO:0048863) | 158 | 6 | 0.29 | 20.44 | 5.40E-07 |
| Regulation of Cell Development (GO:0060284) | 946 | 11 | 1.76 | 6.26 | 8.47E-07 |
| Multicellular Organismal Reproductive Process (GO:0048609) | 826 | 10 | 1.53 | 6.52 | 2.08E-06 |
| Multicellular Organism Reproduction (GO:0032504) | 840 | 10 | 1.56 | 6.41 | 2.41E-06 |
| Regulation of Cell Differentiation (GO:0045595) | 1814 | 14 | 3.37 | 4.15 | 2.50E-06 |
| Reproductive Process (GO:0022414) | 1426 | 11 | 2.65 | 4.15 | 4.10E-05 |
| Reproduction (GO:0000003) | 1429 | 11 | 2.65 | 4.14 | 4.18E-05 |
| Multi-organism Reproductive Process (GO:0044703) | 995 | 9 | 1.85 | 4.87 | 7.19E-05 |
| Reproductive Structure Development (GO:0048608) | 424 | 6 | 0.79 | 7.62 | 1.30E-04 |
| Reproductive System Development (GO:0061458) | 427 | 6 | 0.79 | 7.56 | 1.35E-04 |
| Developmental Process involved in Reproduction (GO:0003006) | 668 | 7 | 1.24 | 5.64 | 2.13E-04 |
| Stem Cell Development (GO:0048864) | 84 | 3 | 0.16 | 19.23 | 5.61E-04 |
| Gamete Generation (GO:0007276) | 691 | 5 | 1.28 | 3.9 | 8.87E-03 |
| Germ Cell Development (GO:0007281) | 257 | 3 | 0.48 | 6.28 | 1.23E-02 |
| Sexual Reproduction (GO:0019953) | 832 | 5 | 1.55 | 3.24 | 1.85E-02 |
| Leydig Cell Differentiation (GO:0033327) | 11 | 1 | 0.02 | 48.94 | 2.20E-02 |
| Seminiferous Tubule Development (GO:0072520) | 12 | 1 | 0.02 | 44.86 | 2.38E-02 |
| Male Gonad Development (GO:0008584) | 136 | 2 | 0.25 | 7.92 | 2.70E-02 |
| Development of Primary Male Sexual Characteristics (GO:0046546) | 137 | 2 | 0.25 | 7.86 | 2.74E-02 |
| Cellular Process involved in Reproduction in Multicellular Organism (GO:0022412) | 353 | 3 | 0.66 | 4.58 | 2.80E-02 |
| Male Sex Differentiation (GO:0046661) | 158 | 2 | 0.29 | 6.81 | 3.55E-02 |

**Table S4. GO-term over-representation test for 39 target genes of the 7 high-expression miRNAs which are upregulated in KS samples**. Here we shown the Biological processes in which target genes, of the differentially expressed miRNAs, participate according to the analysis of PANtHER Classification System, and that are related to male gonadal development. Data are listed according to statistical significance.

| GO biological process complete | Homo sapiens - REFLIST (20851) | miRNAs Target Input | miRNAs Target Input (expected) | miRNAs Target Input (fold Enrichment) | miRNAs Target Input (raw P-value) |
|---|---|---|---|---|---|
| Positive regulation of cell population proliferation (GO:0008284) | 921 | 19 | 3.05 | 6.23 | 1.15E-10 |
| Regulation of cell differentiation (GO:0045595) | 1844 | 24 | 6.1 | 3.93 | 2.50E-09 |
| Developmental process (GO:0032502) | 5900 | 43 | 19.52 | 2.2 | 7.18E-09 |
| Regulation of cell population proliferation (GO:0042127) | 1642 | 22 | 5.43 | 4.05 | 8.65E-09 |
| cell differentiation (GO:0030154) | 3732 | 32 | 12.35 | 2.59 | 5.54E-08 |
| cell population proliferation (GO:0008283) | 495 | 12 | 1.64 | 7.33 | 9.07E-08 |
| cellular developmental process (GO:0048869) | 3825 | 32 | 12.66 | 2.53 | 1.00E-07 |
| cell division (GO:0051301) | 503 | 9 | 1.66 | 5.41 | 4.52E-05 |
| reproductive process (GO:0022414) | 1437 | 15 | 4.76 | 3.15 | 6.23E-05 |
| reproduction (GO:0000003) | 1440 | 15 | 4.77 | 3.15 | 6.38E-05 |
| regulation of cell development (GO:0060284) | 964 | 12 | 3.19 | 3.76 | 7.50E-05 |
| gonad development (GO:0008406) | 214 | 6 | 0.71 | 8.47 | 8.72E-05 |
| reproductive structure development (GO:0048608) | 431 | 8 | 1.43 | 5.61 | 9.63E-05 |
| male gonad development (GO:0008584) | 135 | 5 | 0.45 | 11.19 | 9.95E-05 |
| development of primary sexual characteristics (GO:0045137) | 220 | 6 | 0.73 | 8.24 | 1.01E-04 |
| reproductive system development (GO:0061458) | 435 | 8 | 1.44 | 5.56 | 1.02E-04 |
| development of primary male sexual characteristics (GO:0046546) | 136 | 5 | 0.45 | 11.11 | 1.03E-04 |
| male sex differentiation (GO:0046661) | 158 | 5 | 0.52 | 9.56 | 2.03E-04 |
| Gamete generation (GO:0007276) | 693 | 9 | 2.29 | 3.92 | 4.81E-04 |

**Table S5. GO-term over-representation test for 70 target genes of the 20 high-expression miRNAs which are downregulated in KS samples.** Here we shown the Biological processes in which target genes, of the differentially expressed miRNAs, participate according to the analysis of PANtHER Classification System, and that are related to male gonadal development. Data are listed according to statistical significance.

| Patient | Age | Sample | % of seminiferous tubules with spermatogenesis | % of st with only-Sertoli cells | % of sclerotic seminiferous tubules |
|---|---|---|---|---|---|
| 1 | 36 | KLI1 | 1% | 9% | 90% |
| | | KLI_2 | 1% | 9% | 90% |
| 2 | 35 | KLI_3 | 1% | 9% | 90% |
| | | KLI_4 | 5% | 5% | 90% |
| 3 | 33 | KLI_5 | 1% | 9% | 90% |
| | | KLI_6 | 1% | 9% | 90% |
| 4 | 36 | KLI_7 | 5% | 20% | 75% |

**Table S6. Histological characteristics of the samples**. Summary of characteristics of samples derived from each patient including patient´s age.

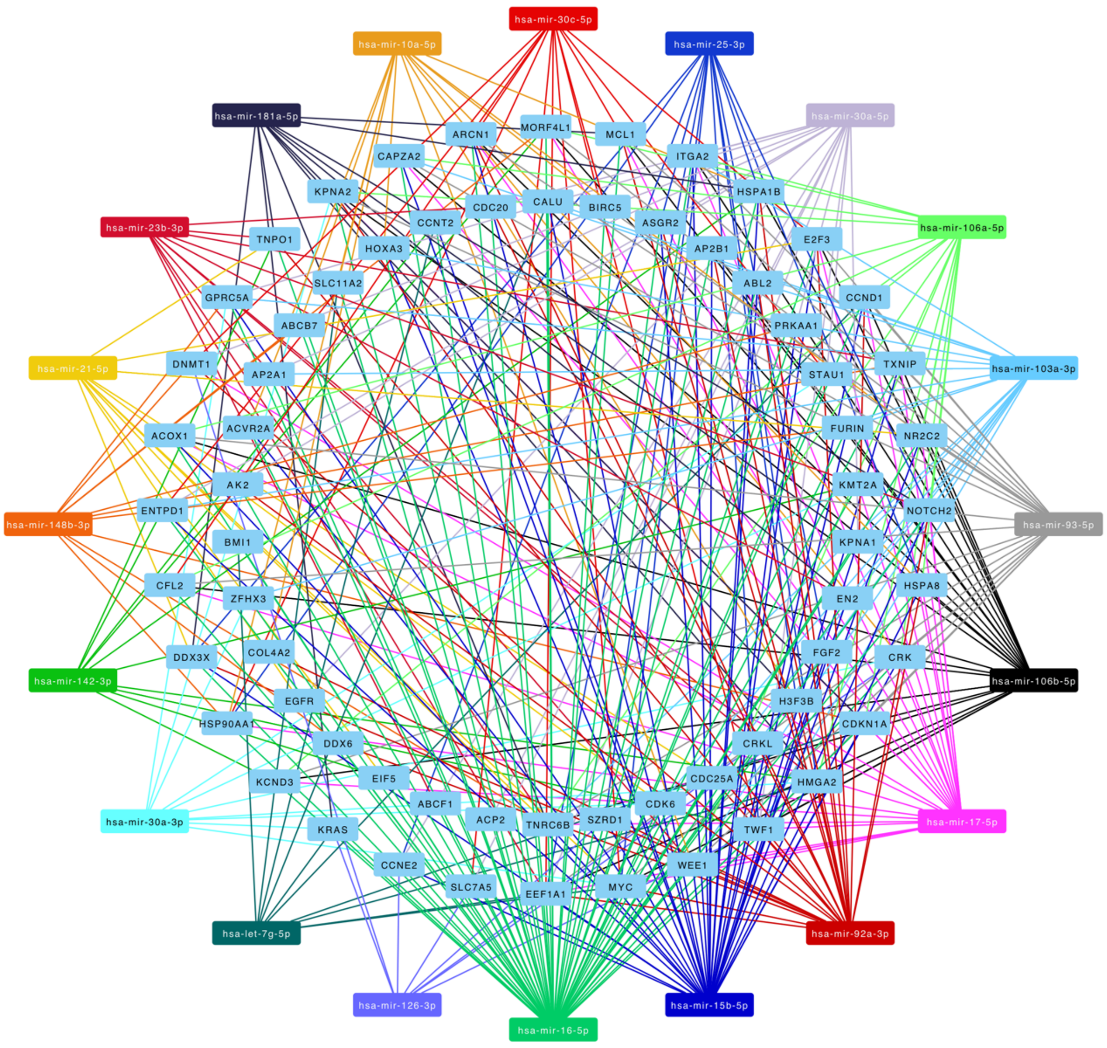


**Figure S1. Supplementary data. miRnet interactome network for the high-expression, downregulated miRNAs in the KS sample subset.** Interaction between highly expressed downregulated miRNAs (rectangles), in the Klinefelter sample tissues with target genes (light blue rectangles).

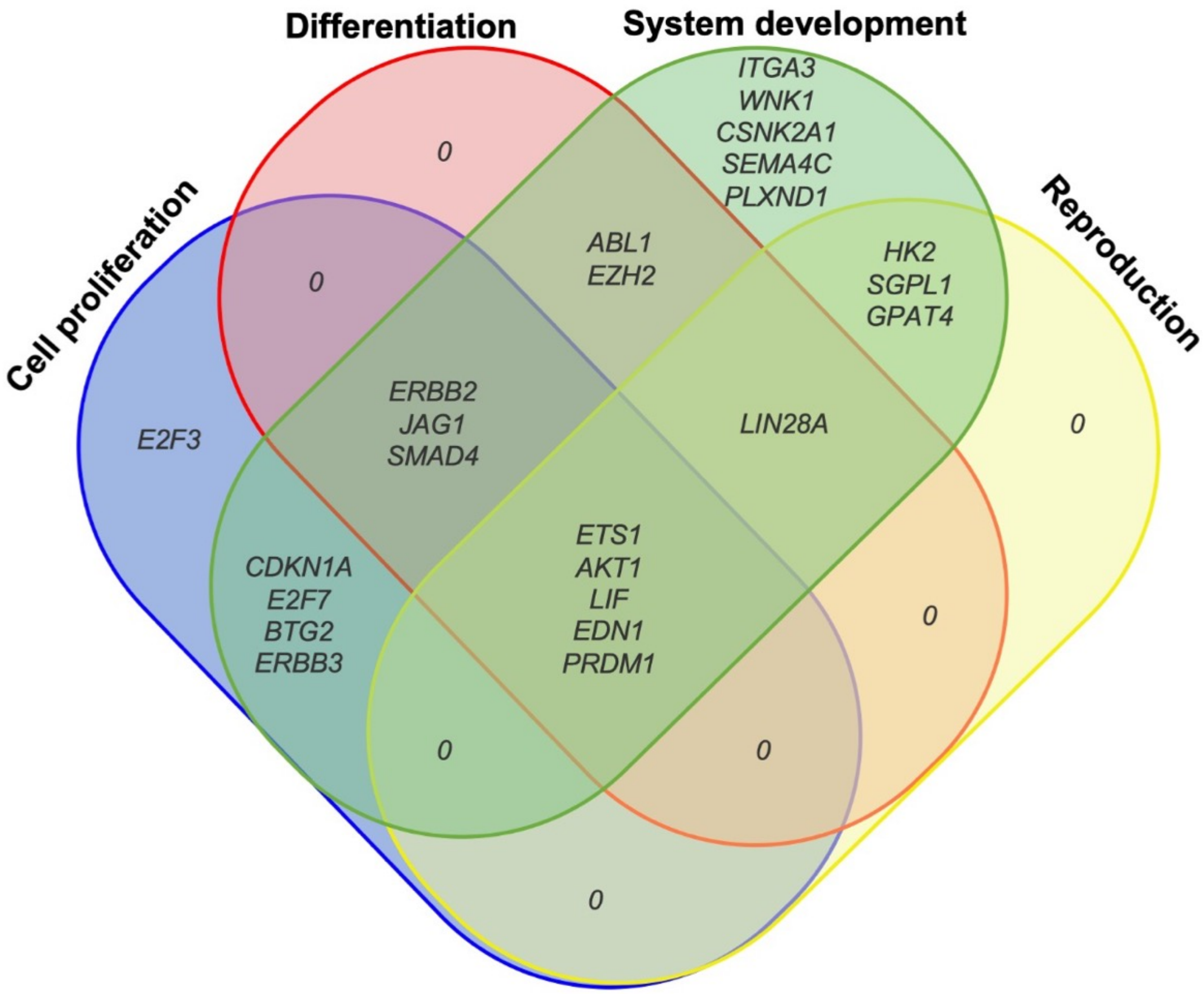


**Figure S2. Supplementary data. Venn diagram of target genes.** This diagram shows the interaction of the target genes of the upregulated miRNAs involved in different cell functions: differentiation, cell proliferation, system development, and reproduction.
